# Laparoscopic Cholecystectomy Performed by Residents: A Retrospective Study on 569 Patients

**DOI:** 10.1155/2014/912143

**Published:** 2014-01-02

**Authors:** Dario Pariani, Stefano Fontana, Giorgio Zetti, Ferdinando Cortese

**Affiliations:** Azienda Ospedaliera Ospedale di Circolo di Busto Arsizio, Presidio Ospedaliero di Saronno, U.O. Chirurgia Generale e Toracica, Piazzale Borella 1, 21047 Saronno, Italy

## Abstract

*Introduction*. Aim of this study was to evaluate the safety of laparoscopic cholecystectomy performed by residents. *Materials and Methods*. We retrospectively reviewed 569 elective laparoscopic cholecystectomies. *Results*. Duration of surgery was 84 ± 39 min for residents *versus*  66 ± 47 min for staff surgeons, *P* < 0.001. Rate of conversion was 3.2% for residents *versus* 2.7% for staff surgeons, *P* = 0.7. There was no difference in the rates of intraoperative and postoperative complications for residents (1.2% and 3.2%) *versus* staff surgeons (1.5% and 3.1%), *P* = 0.7 and *P* = 0.9. Postoperative hospital stay was 3.3 ± 1.8 days for residents *versus*  3.4 ± 3.2 days for staff surgeons, *P* = 0.6. One death in patients operated by residents (1/246) and one in patients operated by staff surgeons (1/323) were found, *P* = 0.8. No difference in the time to return to normal daily activities between residents (11.3 ± 4.2 days) and staff surgeons (10.8 ± 5.6 days) was found, *P* = 0.2. Shorter duration of surgery when operating the senior residents (75 ± 31 minutes) than the junior residents (87 ± 27 minutes), *P* = 0.003. *Conclusion*. Laparoscopic cholecystectomy performed by residents is a safe procedure with results comparable to those of staff surgeons.

## 1. Introduction

Since Philippe Mouret performed the first laparoscopic cholecystectomy in 1987 [1], considerable progress has been made in the field of surgical instruments and equipment, and a great deal of experience in performing the laparoscopic cholecystectomy was acquired around the world. One of the great advantages of laparoscopy is the possibility for the entire surgical team to see with the eyes of the surgeon. For the surgeon in training, this is an important educational opportunity than the open surgery in which, in some steps of the operation, his vision is severely restricted. Despite this advantage, there are some limitations of the laparoscopic surgery which are represented by the lack of tactile feedback, 2-dimensional vision, limited degree of movement of the instruments, and loss of natural hand-eye coordination. The teaching of laparoscopic surgery should be based not just on knowledge of the anatomy and the steps of operation but also on the learning of gestures and tricks of surgical technique which in some cases may be different from the laparotomy surgery. The primary aim of our study was to analyze whether the laparoscopic cholecystectomy performed by surgeons in training is a safe procedure by comparing the same operation performed by trainees and staff surgeons. The secondary aim was to analyze the possible differences within the group of surgeons in training with the progress of their learning-curve.

## 2. Materials and Methods

From April 1, 2009 to March 31, 2013 in the Saronno Operative Unit of General and Thoracic Surgery of the Circle Hospital of Busto Arsizio in agreement with the School of Specialization in General Surgery at the University of Milan, 569 elective laparoscopic cholecystectomies were performed for gallbladder symptomatic stones, biliary dyskinesia, and gallbladder polyps. Laparoscopic cholecystectomies performed for acute cholecystitis and operations in which it was necessary to execute an intraoperative cholangiography were not considered in our study, because, in our institution, usually these surgeries are performed by staff surgeons as first operators. During the period covered in our study, a total of 10 residents of the School of Specialization in General Surgery at the University of Milan attended our Operative Unit. Of these residents 5 (junior residents) were in the first four years of their training course, while, 5 (senior residents) were in the last two years of their training course. The operative technique was performed by placing the patient according to the French school, accessing the abdominal cavity through minilaparotomy and Hasson trocar in the periumbilical area, placing the other three trocars according to the French school, and always trying to get the “critical view of safety” [2]. When the first surgeon of the team was a resident, the second surgeon was always a staff surgeon. The data that we have collected for each patient were gender, age, previous abdominal surgery, body mass index (BMI), ASA class, duration of surgery, conversion to laparotomy, intraoperative and postoperative complications, mortality, length of hospital stay, and time to return to normal daily activities. The data were expressed as means ± standard deviations using Student's *t*-test for continuous variables and the *χ* ^2^ test for dichotomous variables. A probability of <0.05 was considered as significant. The statistical analysis was performed by using Microsoft Excel for Windows XP.

## 3. Results

**Figure 1:**
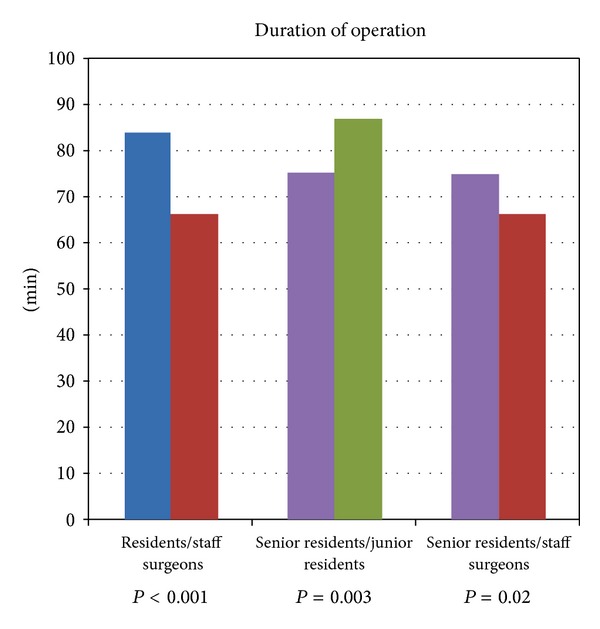
Duration of operation in minutes of elective laparoscopic cholecystectomy in residents *versus* staff surgeons, senior residents *versus* junior residents, and senior residents *versus* staff surgeons.

Of 569 elective laparoscopic cholecystectomies, 246 (43.2%) were performed, as first surgeon, by residents, and 323 (56.8%) were performed, as first surgeon, by staff surgeons. The reasons for operations were gallbladder symptomatic stones in 220 (89.4%) patients operated by residents and in 292 (90.4%) patients operated by staff surgeons, *P* = 0.7; gallbladder polyps in 19 (7.7%) patients operated by residents and in 28 (8.7%) patients operated by staff surgeons, *P* = 0.6; biliary dyskinesia in 7 (2.9%) patients operated by residents and in 3 (0.9%) patients operated by staff surgeons, *P* = 0.08. Of the 246 patients operated by the residents, 147 (59.7%) were female and 99 (40.3%) were males, and of the 323 patients operated by the staff surgeons, 177 (54.7%) were female and 146 (45.3%) were males, *P* = 0.2. The mean age of patients operated by residents was 55 ± 12 years, while that of the patients operated by the staff surgeons was 56 ± 17 years, *P* = 0.4. 57 (23.1%) patients operated by residents and 90 (27.8%) patients operated by the staff surgeons had previously undergone other abdominal operations, *P* = 0.2. In patients operated by residents, the body mass index (BMI) was 27.5 ± 6.9 kg/m^2^, while in patients operated by staff surgeons, BMI was 27.1 ± 7.1 kg/m^2^, *P* = 0.5. The majority of patients operated both by residents and by staff surgeons were ASA class II, and no difference was found in ASA classes I, II, and III, while ASA IV patients were operated only by staff surgeons, *P* = 0.04 (Table 1). The mean duration of operation performed by residents was 84 ± 39 min, while the mean duration of operation performed by staff surgery was 66 ± 47 minutes, *P* < 0.001. Most of the conversions from laparoscopy to laparotomy have been performed due to unclear anatomy and inability to proceed in laparoscopy safely. Among the residents, the percentage of the conversions was 3.2% (8/246) while among the staff surgeons, it was 2.7% (9/323), *P* = 0.7. As shown in detail in Table 2, there were no differences in the percentage of intraoperative complications in patients operated by residents (3/246, 1.2%) *versus* patients operated by staff surgeons (5/323, 1.5%), *P* = 0.7. Also there were no differences in the percentage of postoperative complications in patients operated by residents (8/246, 3.2%) *versus* patients operated by staff surgeons (10/323, 3.1%), *P* = 0.9. In particular, the overall rate of bile duct injury was 0.52% (3/569). The two lesions of the biliary tract highlighted in the postoperative course were of type A according to the classification of Strasberg et al. [2], and they were treated endoscopically through papillosphincterotomy and maintenance of abdominal drainage, while the biliary lesion immediately recognized during surgery was a type D, therefore, after conversion to laparotomy, the bile duct lesion was sutured and a Kher tube was placed. The two bowel injuries that have occurred as a result of caustic damage were sutured during the same operation laparoscopically. The intraoperative bleeding complications were treated by bipolar coagulation in case of bleeding from the liver bed and putting additional clips in case of injury of the cystic artery. In the three cases of postoperative bleeding, the patient was subjected to laparoscopic reoperation that solved the bleeding from the epigastric trocar in one case, while in the other two cases, it was necessary to convert to laparotomy technique to stop the bleeding oozing from the liver bed. A case of death due to acute myocardial infarction in a ASA III cardiac patients was recorded in patients operated by residents (1/246, 0.4%), and one case of death due to stroke was recorded in an ASA IV patient operated by staff surgeons (1/323, 0.3%), *P* = 0.8. The mean postoperative hospital stay of patients operated by residents was 3.3 ± 1.8 days, while the mean postoperative hospital stay of patients operated by staff surgeons was 3.4 ± 3.2 days, *P* = 0.6. No difference was found in the time of return to normal daily activities between patients operated by residents (11.3 ± 4.2 days) and staff surgeons (10.8 ± 5.6 days), *P* = 0.2. The analysis within the group of patients operated on by residents showed that the only statistically significant difference is represented by a shorter duration of operation when operating the senior residents (75 ± 31 minutes) than the junior residents (87 ± 27 minutes), *P* = 0.003. This result, however, must take account of the small numbers of cholecystectomies performed by junior residents (*n* = 79, 32.1%) compared to those performed by senior residents (*n* = 167, 67.9%), *P* = 0.001. The difference in duration of the operation is statistically significant even when comparing the times of the senior residents (75 ± 31 minutes) with the staff surgeons (66 ± 47 minutes), *P* = 0.02 (Figure 1).

## 4. Discussion

Laparoscopic procedures represent a challenge for teaching in the operating room as the experienced surgeon is less likely to intervene during the procedure than during a laparotomy operation. During most of the laparotomy procedures, there is considerable ductility between staff surgeon and resident so that, at the beginning, the younger surgeon can deal with the easier steps of the operation leaving the most difficult passages to the senior surgeon. In laparoscopy, instead interchangeability is more difficult to obtain since the position of the trocars is fixed and very often the change of operator would entail the variation of the position of the surgeons at the operating table. There is a broad consensus among surgeons about the fact that the necessary skills for laparoscopic surgery are partly different from those used in laparotomy surgery [3, 4]. The training in laparoscopic surgery is multifaceted and must include, in addition to familiarity with the laparoscopic instrumentation, also the mastery of basic skills needed to operate safely. A laparoscopic surgeon must learn to operate with long instruments that amplify physiological tremor and are more difficult to control than the tools of traditional surgery. These same tools are limited in their range of motion as inserted in trocars which act as a fulcrum. A further difficulty, in learning of laparoscopic surgery, is represented by the fact that the surgeon must learn to operate in another direction than his vision, watching a monitor that provides two-dimensional images and limits the depth perception. Thanks to an agreement with the School of Specialization in General Surgery at the University of Milan, each resident of our institution has the opportunity to practice on a laparoscopic surgery simulator in order to become familiar with laparoscopic instruments and with the basic steps of the operation. In our Operative Unit, when the first surgeon is a resident, we use a specific checklist in which there are eleven verbal checks between the resident and the staff surgeon that formalize the need for consent of both surgeons before proceeding to the next step of the operation. Furthermore, when the first surgeon is a resident, in our institution, it is required to have a preoperative briefing and a postoperative debriefing with the staff surgeon in order to improve safety and attitudes of the members of the surgical team.

Our study shows that laparoscopic cholecystectomy can be performed by residents with rates of intraoperative and postoperative complications comparable to those of staff surgeons. As expected, our data show that the duration of operation was significantly higher in cholecystectomies performed by residents compared to those performed by staff surgeons (84 *versus* 66 minutes). The same difference in the duration of laparoscopic cholecystectomy was reported by a study published by Böckler et al. [5] although with times higher than what we found (119 minutes for residents *versus* 97 minutes for staff surgeons). The difference in duration of the operation can be explained, in part, as evidenced by other studies in the literature [6, 7], with the lower surgical skill of the residents and in part by the fact that it is often the staff surgeon himself to teach the resident that time should not be a primary concern and that he should always pay maximum attention to what he is doing even in the steps of the operation that may seem simple. Our study shows that the duration of laparoscopic cholecystectomy decreases over the 6 years of training in general surgery with the gradual progress in the learning curve. A similar result was also pointed out by Kauvar et al. [8] whose study showed that the mean duration of laparoscopic cholecystectomy performed by residents in the first three years of training was 88 minutes, while in the last 2 years of training, it was 73 minutes. A study published by Hobbs et al. [9] demonstrated that the intraoperative complications during laparoscopic cholecystectomy occur in 1.37% of patients operated by surgeons with an experience of less than 50 cholecystectomies, while this rate drops to 0.8% when the surgeon has performed at least 300 procedures of laparoscopic cholecystectomy. Contrary to what Hobbs reported, we found no difference in intraoperative complications of laparoscopic cholecystectomies performed by residents and by staff surgeons. Our same result was also shown by Koulas et al. [6] who in 2006 published a study comparing 445 patients undergoing laparoscopic cholecystectomy and operated by surgeons in training with 925 patients undergoing laparoscopic cholecystectomy and operated by staff surgeons. The intraoperative complications including bile duct injuries, bleeding, and bowel injuries were found to be rare with no statistically significant differences between the two groups. Overall, considering the total number of lesions of the biliary tract in both groups, the rate is 0.52%, which is comparable with that reported by most of the studies in the literature [10, 11] where the percentage of biliary injury during laparoscopic cholecystectomy ranges from 0.47% to 0.62%. In our study, we recorded 2 cases of postoperative deaths for medical causes (myocardial infarction and stroke) with a rate of 0.35% (2/569), and this finding is comparable to those reported in the literature where studies [12, 13], regarding a great number of patients who undergone laparoscopic cholecystectomy, show a mortality rate from 0.3 to 0.5%. The overall conversion rate was 2.9% (17/569), and no difference was found between the rate of conversion in the operations performed by residents compared to those carried out by staff surgeons (3.2% *versus* 2.7%). This result is in line with those reported in most of the studies [14] in the literature in which the percentage of conversion varies from 2 to 15%. In this regard, it is interesting to note that even the conversion rate is similar and not significant (*P* = 0.7) for both junior and senior residents (3.6% *versus* 3.1%), and this result contrasts with what has been reported by Kauvar et al. [8] that in their study shows that the conversion rate to laparotomy is significantly greater in the operations performed by residents in their last years of training than that in the operations performed by residents in their first three years of training (8.4% *versus* 3.7%). We found no difference between the rate of postoperative complications of the two groups (3.2% *versus* 3.1%) and the data we have obtained are comparable to those already published by Koulas et al. [6] who record a complication rate of 2.7% in laparoscopic cholecystectomies performed by surgeons in training *versus* 3.7% in the laparoscopic cholecystectomies performed by trained surgeons. In our study, the mean postoperative hospital stay in the two groups of patients is comparable (3.3 *versus* 3.4 days), and this result was also highlighted in the study of Böckler et al. [5] where, however, postoperative hospital stay is detected slightly longer (5 days for the patients operated by staff surgeons and 6 days for patients operated by residents) than that we have found. In a recent paper comparing 150 laparoscopic cholecystectomies performed by trained surgeons and 297 laparoscopic cholecystectomies performed by surgeons in training, Sanjay et al. [15] showed that, in the first case, the mean postoperative hospital stay was 1 day while in the second case it was 2 days.

## 5. Conclusion

Despite the limits of a retrospective analysis, our study shows that laparoscopic cholecystectomy performed by residents is a safe procedure and not burdened by an increase in the rates of complications, conversion to laparotomy, mortality, and length of hospital stay, provided that there is an adequate supervision by an experienced surgeon and when the operation is carried out within a scenario in which the steps of the operation are clear and standardized.

## Figures and Tables

**Table 1:** ASA (American Society of Anaesthesiologists) score of the patients operated by residents and staff surgeons.

ASA score	ASA I	ASA II	ASA III	ASA IV
Residents	74 (30%)	140 (57%)	32 (13%)	0 (0%)
Staff surgeons	90 (28%)	189 (59%)	39 (12%)	5 (1%)
*P* value	*0.9 *	*0.7 *	*0.8 *	*0.04 *

**Table 2:** Intraoperative and postoperative complications of the patients operated by residents and staff surgeons.

Complications	Residents (*n* = 246)	Staff surgeons (*n* = 323)	*P* value
Intraoperative			
Bowel injuries	0	2	
Biliary injuries	1	0	
Bleeding			
Liver bed	1	2	
Cystic artery	1	1	

Total	3	5	*0.7 *

Postoperative			
Biliary leakage	0	2	
Bleeding			
Epigastric trocar	1	0	
Liver bed	1	1	
Infections	6	7	

Total	8	10	*0.9 *
